# Robot-assisted repair of delayed traumatic diaphragmatic hernia: a case report

**DOI:** 10.1093/jscr/rjae197

**Published:** 2024-04-01

**Authors:** Paxton Holder, Mohamed-Aly Bakeer

**Affiliations:** Department of Clinical Affairs, Edward Via College of Osteopathic Medicine, Monroe, LA, United States; The Surgery Clinic of Northeast Louisiana and the Department of Surgery at Saint Francis Medical Center, Monroe, LA, United States

**Keywords:** traumatic diaphragmatic hernia, robot-assisted surgery, abdominal trauma

## Abstract

Traumatic diaphragmatic hernia is a sign of severe thoracoabdominal trauma that is often difficult to detect because of nonspecific presenting symptoms, delayed presentation, and distracting injuries. Diagnosis depends on imaging and a high degree of suspicion in patients who present with respiratory or gastrointestinal symptoms after trauma, and prompt surgical repair is required. This case reviews a patient who presented to the emergency department with burning epigastric pain radiating to the left chest and hematemesis ~1 month after sustaining a blunt abdominal injury. Imaging studies revealed a substantial portion of the gastric body in the left hemithorax. Robot-assisted reduction of the stomach was performed followed by repair with tension-free primary closure without mesh reinforcement and gastropexy. The patient was monitored for return of bowel function and discharged upon recovery. This case report highlights the diagnostic challenges of traumatic diaphragmatic hernia and the benefit of robot-assisted repair.

## Introduction

### Background

Diaphragmatic rupture is a rare complication in cases of thoracoabdominal trauma that involves a rapid increase of the pressure gradient across the pleuroperitoneal membrane. This complication occurs in ~5% of abdominal traumas [1]. The reported incidence rate varies, but it appears that rupture is consistently associated with penetrating trauma (10%–19% [1]; 67% [2]) more often than blunt trauma (5% [1]; 33% [2]). A tremendous amount of force must be sustained to produce a defect in the diaphragm, and the overall mortality rate, ranging from 18% (blunt injury) to 40% (penetrating injury), is often because of collateral injuries [3].

The clinical manifestations of traumatic diaphragmatic hernia (TDH) can be classified as acute, latent, or obstructive [3]. Rashid *et al*. [4] suggested that up to 30% of TDH have a delayed presentation. It has been proposed that the diaphragm remains intact initially, but an inflammatory process weakens it over the ensuing days until it finally degrades [5]. Upon diagnosis, the defect must be surgically repaired.

### Imaging

Considering the mortality rate associated with TDH, it is important to be familiar with the signs that may present on imaging for efficient diagnosis. Even though CT can be an effective means of diagnostic imaging, 12%–63% of diagnoses are initially missed [6].

Table 1 describes the frequency [6] and helpful descriptions [7] of common imaging signs consistent with TDH.

**Table 1:** Table reporting frequency and descriptions of common imaging signs consistent with TDH.

**Frequency of imaging signs (6)**	**Description of imaging signs (7)**
Diaphragmatic discontinuity (95.7%)	Visualization of the defect and the free edge of the ruptured diaphragm with or without thickening of the free edge.
Thickened diaphragm (69.6%)	The affected portion can be compared with the contralateral hemidiaphragm for signs of thickening because of post-traumatic edema and hemorrhage.
Intrathoracic herniation of abdominal contents (65.2%)	Because of the pressure gradient across the thoracic and abdominal cavities.
“Dependent viscera” sign (56.5%)	Abdominal viscera normally do not contact the posterior chest wall. Without the diaphragm to maintain this partition, the viscera are allowed to move posteriorly (in a “dependent position”) when supine.
“Dangling diaphragm” sign (56.5%)	The free edge of the disrupted diaphragm curls inward and away from the chest wall forming a “comma-shaped fragment” even if the diaphragmatic discontinuity sign is not present.
“Collar” sign (43.5%)	A luminal constriction of the herniated contents at the site of the defect.

## Case report

A 61-year-old male presented to the emergency department with dyspnea, chest pain, and left shoulder pain after being ejected from an off-road vehicle. A comprehensive physical exam revealed a stable patient in no acute distress with tachycardia and hypertension. Tenderness to palpation was appreciated at the left anterior chest wall and at the midline thoracic spine. Breath sounds were present except at the left lower base. CT revealed a left hemopneumothorax, fractures of ribs 2–12 on the left, fracture of the left transverse process at T3, and fracture of the blade of the left scapula. A chest tube drained 1 l of blood, and the patient was placed in a shoulder immobilizer. The patient was discharged 1 week later. He remained stable at his follow-up appointment 2 weeks after discharge.

**Figure 1:**
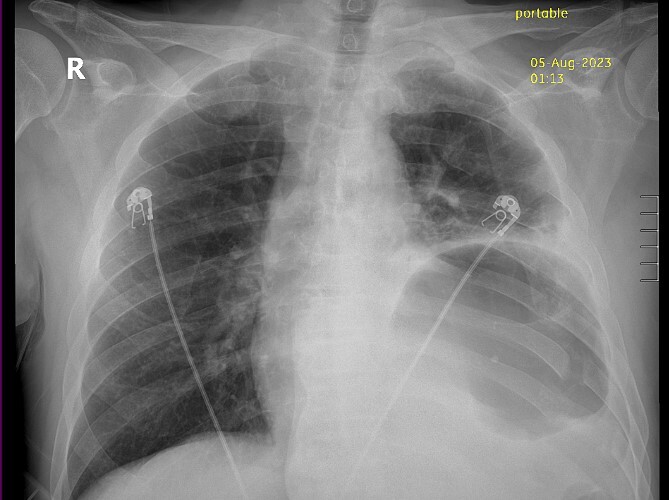
Chest X-ray showing gastric body in the left hemithorax.

**Figure 2:**
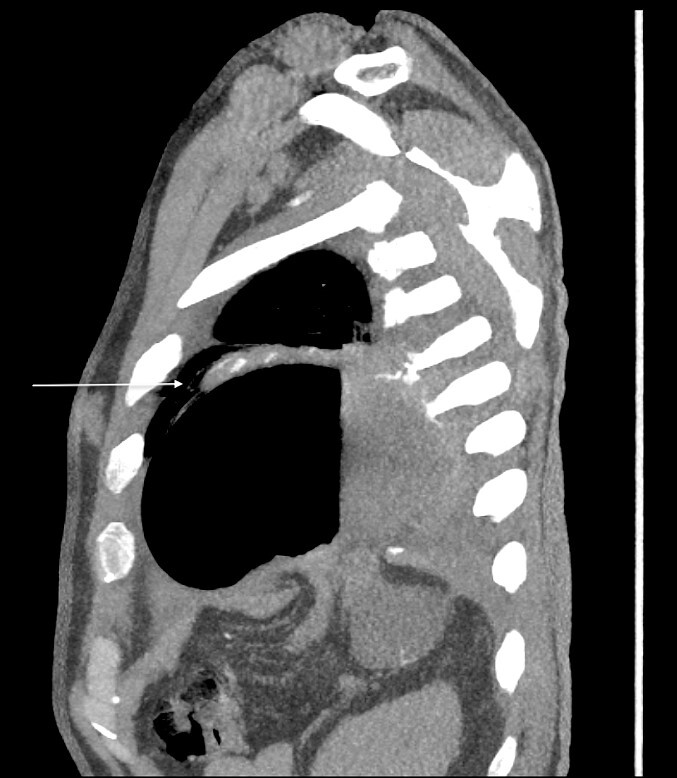
Sagittal CT showing gastric body herniating through diaphragmatic defect and the “dangling diaphragm” sign (arrow).

**Figure 3:**
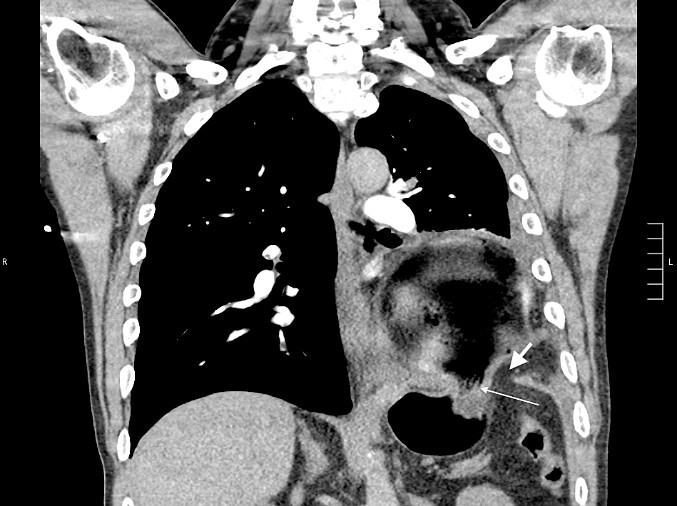
Coronal CT showing the gastric body herniating through diaphragmatic defect, discontinuity of the diaphragm (thick arrow), and the “collar” sign (thin arrow).

**Figure 4:**
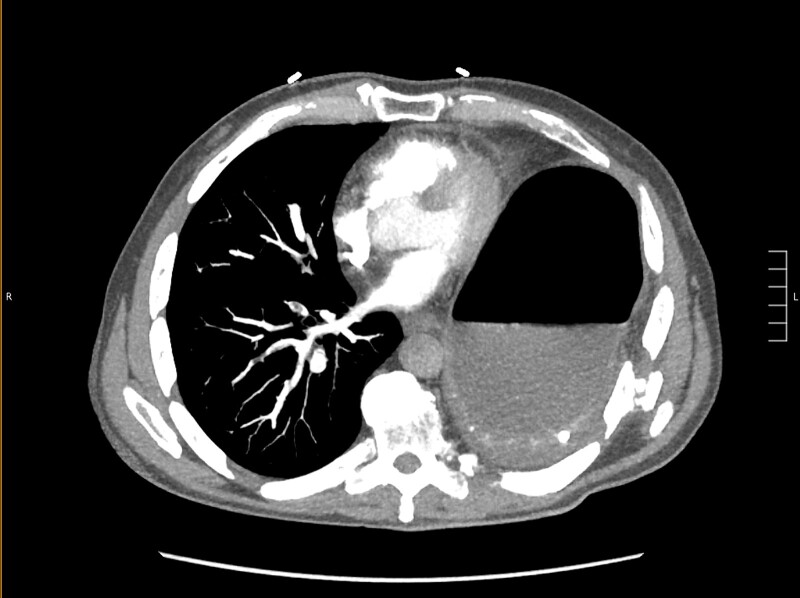
Axial CT showing previously fractured ribs, the “dependent viscera” sign, and thoracic fluid abutting abdominal viscera.

Thirty-seven days after the initial visit, the patient returned to the emergency department with burning epigastric pain that radiated to the left upper chest and hematemesis. Vital signs revealed tachycardia and hypertension. A comprehensive physical exam was significant for decreased breath sounds at the left base and tenderness in the left upper quadrant without signs of peritonitis. Pertinent laboratory findings showed leukocytosis with a left shift and thrombocytosis. A chest X-ray showed a significant portion of the gastric body in the left hemithorax (Fig. 1). A pulmonary CT angiogram found segmental and subsegmental pulmonary emboli in both lungs with no evidence of right heart strain and a left pleural effusion. A left diaphragmatic hernia with the “dangling diaphragm” sign can be seen on the sagittal view (Fig. 2). The coronal view shows discontinuity of the diaphragm and the “collar” sign (Fig. 3); the axial view shows the “dependent viscera” sign and thoracic fluid abutting abdominal viscera (Fig. 4).

**Figure 5:**
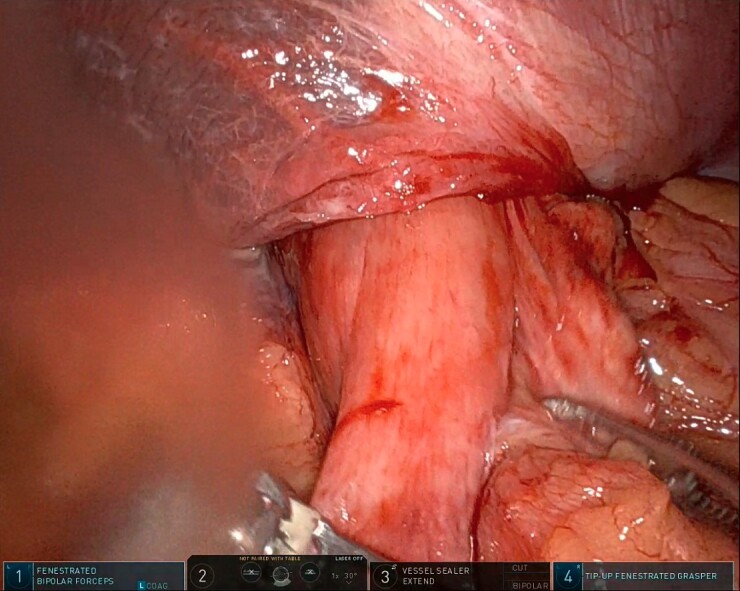
Intraoperative photo showing a large portion of the stomach herniating through the diaphragmatic defect. This photo shows the small portion that remained in the abdominal cavity.

**Figure 6:**
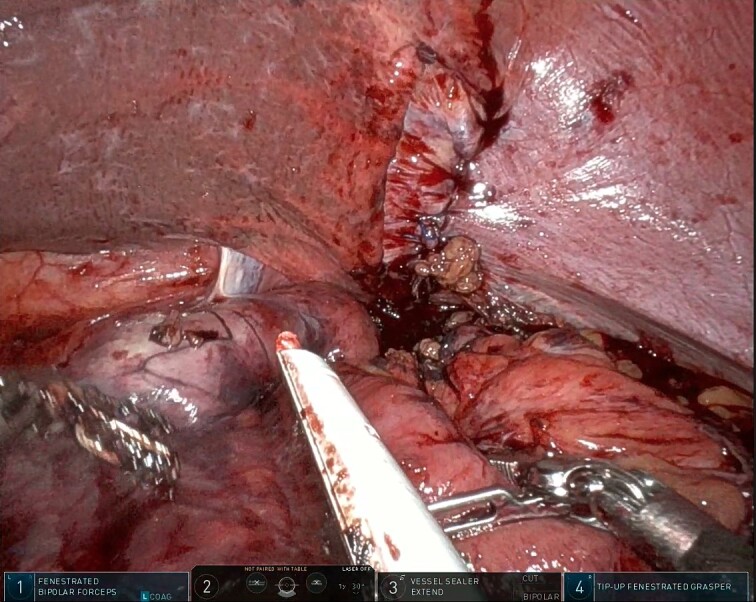
Intraoperative photo showing the layered closure and plication of the diaphragmatic defect. Note the dusky areas of mucosa (bottom left) suggesting compromised perfusion of the herniated viscus.

**Figure 7:**
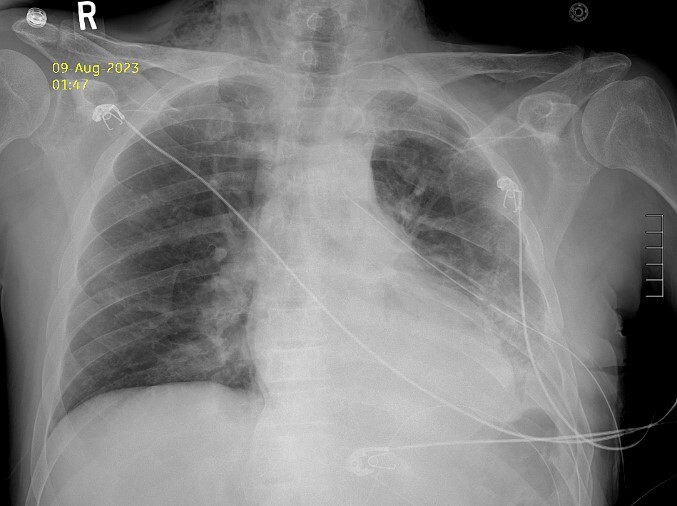
Postoperative Day 2 chest X-ray showing reduced diaphragmatic hernia and resolving pleural effusion.

After the pulmonary emboli were medically optimized, robot-assisted surgical repair was successfully completed with the da Vinci Xi system (Intuitive Surgical Inc.). Upon insertion of the camera and trocars, the stomach was noted to be contained within an 8-cm diaphragmatic defect. Gentle traction was used to reduce the stomach with some difficulty because of the significant portion of the gastric body that had herniated (Fig. 5) as well as adhesions that had formed within the thoracic cavity. Upon reduction of the stomach, the TDH was reapproximated in two layers with the second layer plicating the diaphragm because of the noted laxity (Fig. 6). A gastrostomy tube was placed, and a gastropexy was performed. Finally, a thoracostomy was performed at the left fifth intercostal space, and a chest tube was placed in the left pleural cavity and connected to vacuum suction. The patient tolerated the procedure well, and serial chest X-rays were taken to monitor recovery of the pneumothorax and pleural effusion (Fig. 7). The chest tube was removed, and the patient was discharged 10 days later in stable condition with instructions for a soft diet. The patient was well at the 2-month follow-up visit, and the gastrostomy tube was removed.

## Discussion

A limited number of reports document robot-assisted repair of TDH [8]. Our case represents the feasibility of robot-assisted repair of TDH in the delayed presentation. Considering the number of delayed TDH presentations, it is important to consider the benefits of robot-assisted repair. This approach allows operation in otherwise difficult anatomical spaces because of the high degree of maneuverability and 3D view. In general, minimally invasive surgical technique such as this is associated with fewer complications, a shorter hospital stay, and better esthetic results [9]. Many of these benefits were appreciated in this case as well.

The presence of diaphragmatic rupture has been described as a “marker of severe trauma,” but it is a unique sign because of the frequent difficulty in detection [5]. Previous reports have suggested adopting a high degree of suspicion for TDH if a patient with a history of trauma is readmitted with gastrointestinal symptoms [4] or if any of the associated signs are found on imaging [7]. Our case was a delayed presentation with a relatively stable physical exam. Several specific signs consistent with previous studies were present on imaging in this case, underscoring the importance of being familiar with these signs as the anatomy can be significantly distorted.

## Conclusions

This report showcases the subtlety with which a TDH can present in the delayed setting, highlighting signs that may assist in a timely diagnosis. Our patient initially presented with nonspecific signs that were not necessarily gastrointestinal in nature along with multiple distracting injuries. Initial imaging did not reveal signs of diaphragmatic injury at the time of admission or upon retrospective review. More sensitive imaging modalities might have suggested diaphragmatic injury, but no findings indicated the unnecessary costs and/or radiation. Additionally, an invasive diagnostic procedure might have resulted in an earlier diagnosis and repair; however, considering the patient’s stable exam, surgical exploration was not warranted. At the time of readmission, symptoms stemmed from a gastrointestinal cause and imaging was diagnostic. Considering these findings, we believe care was optimal.
